# Supplementary material for: Recognition of phylogenetically diverse pathogens through enzymatically amplified recruitment of RNF213
Source: EMBO Rep. 2024 Oct 7;25(11):4979–5005. doi: 10.1038/s44319-024-00280-w (PMC11549300; doi:10.1038/s44319-024-00280-w)

Table of content

	page
Appendix Table S1	2
Appendix Table S2	4
Appendix Figure S1	6

Appendix Table S1

Maximum likelihood analysis of positive selection among simian primate RNF213 sequences using the codeml algorithm. Shown are the results from a full-length alignment (top row), and from 6 alignments that each represent a segment of RNF213 that is free from recombination according to the GARD algorithm. For each alignment, we present the overall 'average' dN/dS (codeml model 0), the p-values for two complementary tests for positive selection (model 8 versus 8a, and model 8 versus 7), and predictions from model 8 of the proportion and dN/dS of rapidly evolving sites. Our findings are robust to the use of different starting parameters: we show results for four parameter combinations: initial_omega=0.4 or 3, and codon_model=2 or 3.

Alignment	Codon model	Initial omega	Segment start nucleotide position in alignment	Segment end nucleotide position in alignment	Alignment length (codons)	Number of sequences	Overall dN/dS (model 0)	p-value model 8 versus 8a	p-value model 8 versus 7	Percent of sites under positive selection	Estimated dN/dS of sites under positive selection	Number of sites under positive selection (BEB posterior probability >=0.9)
**Parameter set 1: codon model 2, initial omega 0.4**												
full length	2	0,4			5241	24	0,41	8,1E-42	8,0E-50	4,2	3,3	59
GARD segment 1	2	0,4	1	1152	384	22	0,86	3,8E-05	7,4E-05	17,1	2,6	3
GARD segment 2	2	0,4	1156	1827	224	23	0,85	3,5E-16	3,6E-16	7,6	6,3	11
GARD segment 3	2	0,4	1831	4260	810	24	0,79	2,9E-16	2,8E-15	11,4	3,3	42
GARD segment 4	2	0,4	4264	10791	2176	24	0,24	1,1E-09	2,9E-12	2,9	2,8	18
GARD segment 5	2	0,4	10795	14217	1141	24	0,44	3,8E-10	3,9E-10	3,7	3,7	13
GARD segment 6	2	0,4	14221	15723	501	24	0,30	1	1			
**Parameter set 2: codon model 2, initial omega 3**												
full length	2	3			5241	24	0,41	8,1E-42	8,0E-50	4,2	3,3	59
GARD segment 1	2	3	1	1152	384	22	0,86	3,8E-05	7,4E-05	17,1	2,6	3
GARD segment 2	2	3	1156	1827	224	23	0,85	3,5E-16	3,6E-16	7,6	6,3	11
GARD segment 3	2	3	1831	4260	810	24	0,79	2,9E-16	2,8E-15	11,4	3,3	42
GARD segment 4	2	3	4264	10791	2176	24	0,24	1,1E-09	2,9E-12	2,9	2,8	18
GARD segment 5	2	3	10795	14217	1141	24	0,44	3,8E-10	3,9E-10	3,7	3,7	13
GARD segment 6	2	3	14221	15723	501	24	0,30	1	1			

Appendix Table S1 (continued)

Alignment	Codon model	Initial omega	Segment start nucleotide position in alignment	Segment end nucleotide position in alignment	Alignment length (codons)	Number of sequences	Overall dN/dS (model 0)	p-value model 8 versus 8a	p-value model 8 versus 7	Percent of sites under positive selection	Estimated dN/dS of sites under positive selection	Number of sites under positive selection (BEB posterior probability >=0.9)
**Parameter set 3: codon model 3, initial omega 0.4**												
full length	3	0,4			5241	24	0,42	2,4E-44	2,7E-52	4,5	3,3	59
GARD segment 1	3	0,4	1	1152	384	22	0,92	5,0E-07	1,7E-06	16,2	3,1	6
GARD segment 2	3	0,4	1156	1827	224	23	0,85	6,0E-17	5,3E-17	7,3	6,7	10
GARD segment 3	3	0,4	1831	4260	810	24	0,87	1,6E-19	1,6E-18	11,2	3,8	34
GARD segment 4	3	0,4	4264	10791	2176	24	0,24	1,5E-09	1,8E-12	3,4	2,5	17
GARD segment 5	3	0,4	10795	14217	1141	24	0,45	6,4E-11	9,1E-11	4,3	3,6	13
GARD segment 6	3	0,4	14221	15723	501	24	0,31	1	1			
**Parameter set 4: codon model 3, initial omega 3**												
full length	3	3			5241	24	0,42	2,4E-44	2,7E-52	4,5	3,3	59
GARD segment 1	3	3	1	1152	384	22	0,92	5,0E-07	1,7E-06	16,2	3,1	6
GARD segment 2	3	3	1156	1827	224	23	0,85	6,0E-17	5,3E-17	7,3	6,7	10
GARD segment 3	3	3	1831	4260	810	24	0,87	1,6E-19	1,6E-18	11,2	3,8	34
GARD segment 4	3	3	4264	10791	2176	24	0,24	1,5E-09	1,8E-12	3,4	2,5	17
GARD segment 5	3	3	10795	14217	1141	24	0,45	6,4E-11	9,1E-11	4,3	3,6	13
GARD segment 6	3	3	14221	15723	501	24	0,31	1	1			

Appendix Table S2

CryoEM data collection and processing, model refinement and validation statistics.

	RNF213 (EMDB 19653) (PDB 8S24)
**Data collection and processing**	
Microscope	Titan Krios
Specimen temperature (K)	~80
Voltage (kV)	300
Camera	Falcon 4i
Energy filter	Selectris X
Energy filter slit width (eV)	10
Electron fluence (e–/Å^2)	29.8
Electron flux (e^-/pix/s)	5.2
Exposure time (s)	4.86
Magnification	130,000×
Pixel size (Å)	0.921
Defocus range (μm)	0.5 – 3.0
Average defocus (μm)	2.0
Total number of movies	7,022
**Data processing**	
Initial number of particle images	1,010,981
Final number of particle images	143,490
Particle box size (pixels)	512×512
Symmetry imposed	C1
Map resolution (Å)	3.0 Å (0.143 global FSC cutoff)
Local resolution range (Å)	2.5 - 4.5 Å
**Refinement**	
Initial model used (PDB code)	N/A
Refinement package	COOT, Phenix
Model resolution (Å)	3.0 Å (0.5 map-model FSC cutoff)
Map sharpening *B* factor (Å^2)	30 Å^2

Model composition	
Non-hydrogen atoms	35,357
Protein residues	4387
Ligands	3 (Mg, Zn, ATP)
Molecular weight (kDa, incl H)	503
B factors (Å^2)	
Protein	90
Ligand	60
RMS deviations	
Bond lengths (Å)	0.01
Bond angles (°)	0.5
Validation	
MolProbity score	1.7
Clashscore	8.9
Poor rotamers (%)	0.7
Cβ deviations (%)	0.0
CaBLAM outliers (%)	1.7
Ramachandran plot	
Favored (%)	96.6
Allowed (%)	3.2
Outliers (%)	0.2

Appendix Figure S1

Confocal micrographs of RNF213^{KO} MEFs stably expressing the indicated RNF213 variants. Cells were fixed at 3.5h post-infection with mCherry-expressing *S.* Typhimurium, 6h post-infection with mCherry-expressing *L. monocytogenes* ΔActA and 24h post-infection with Tomato-expressing *T. gondii* Type I strain RH. Scale bar, 10µm.

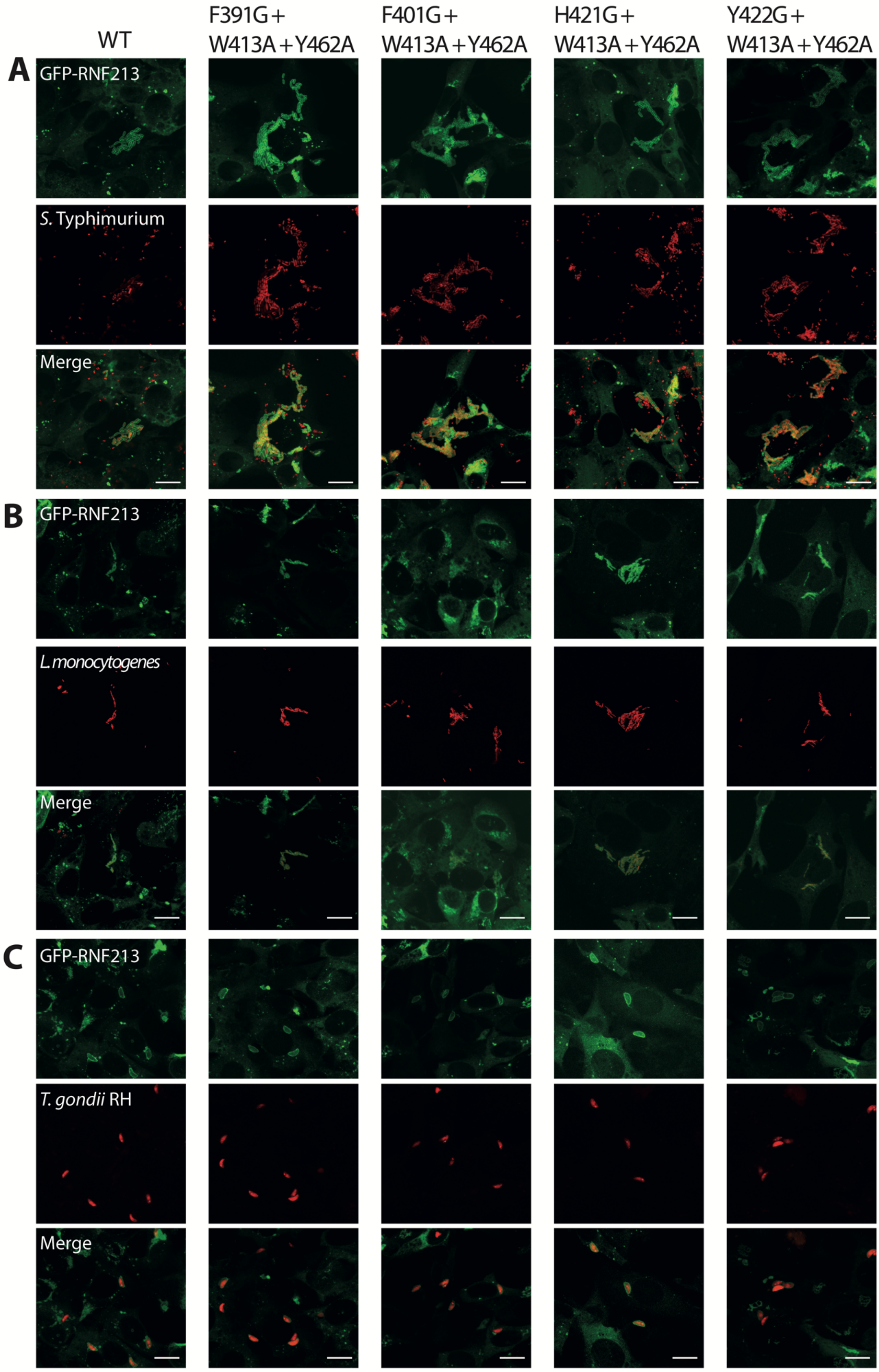

Supplement: Supplementary file 1 — Appendix [file 44319_2024_280_MOESM1_ESM.pdf]
