# Supplementary figures and images for: Recognition of phylogenetically diverse pathogens through enzymatically amplified recruitment of RNF213
Source: EMBO Rep. 2024 Oct 7;25(11):4979–5005. doi: 10.1038/s44319-024-00280-w (PMC11549300; doi:10.1038/s44319-024-00280-w)

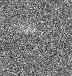

Supplement: Supplementary file 7 — EV Figures source data [file 44319_2024_280_MOESM7_ESM.zip › Source Data_EV figuresAndAppendix/EV2/Parasite17_231204_244_30MIN_1min_2002.nd2 - 231204_244_30MIN_1min_2002.nd2 (series 3)-1.tif]

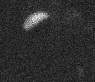

Supplement: Supplementary file 7 — EV Figures source data [file 44319_2024_280_MOESM7_ESM.zip › Source Data_EV figuresAndAppendix/EV2/Parasite22_231212_244_19MIN_1min_.nd2 - 231212_244_19MIN_1min_.nd2 (series 2)-1.tif]

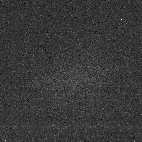

Supplement: Supplementary file 7 — EV Figures source data [file 44319_2024_280_MOESM7_ESM.zip › Source Data_EV figuresAndAppendix/EV2/Parasite1_from video on manuscript_211013_GFPRNF_RHtomato_1hmin_30s008-1.tif]

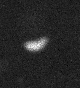

Supplement: Supplementary file 7 — EV Figures source data [file 44319_2024_280_MOESM7_ESM.zip › Source Data_EV figuresAndAppendix/EV2/Parasite25_231212_244_19MIN_1min_.nd2 - 231212_244_19MIN_1min_.nd2 (series 3)-1.tif]

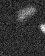

Supplement: Supplementary file 7 — EV Figures source data [file 44319_2024_280_MOESM7_ESM.zip › Source Data_EV figuresAndAppendix/EV2/Parasite6_231129_23MIN_1min.nd2 - 231129_23MIN_1min.nd2 (series 1)-1.tif]

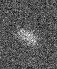

Supplement: Supplementary file 7 — EV Figures source data [file 44319_2024_280_MOESM7_ESM.zip › Source Data_EV figuresAndAppendix/EV2/Parasite11_231129_24MIN_1min_2.nd2 - 231129_24MIN_1min_2.nd2 (series 4)-1.tif]

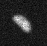

Supplement: Supplementary file 7 — EV Figures source data [file 44319_2024_280_MOESM7_ESM.zip › Source Data_EV figuresAndAppendix/EV2/Parasite7_231129_23MIN_1min.nd2 - 231129_23MIN_1min.nd2 (series 2)-1.tif]

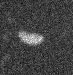

Supplement: Supplementary file 7 — EV Figures source data [file 44319_2024_280_MOESM7_ESM.zip › Source Data_EV figuresAndAppendix/EV2/Parasite15_231204_244_30MIN_1min_2002.nd2 - 231204_244_30MIN_1min_2002.nd2 (series 2)-1.tif]

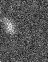

Supplement: Supplementary file 7 — EV Figures source data [file 44319_2024_280_MOESM7_ESM.zip › Source Data_EV figuresAndAppendix/EV2/Parasite19_231204_244_30MIN_1min_2002.nd2 - 231204_244_30MIN_1min_2002.nd2 (series 4)-1.tif]

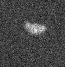

Supplement: Supplementary file 7 — EV Figures source data [file 44319_2024_280_MOESM7_ESM.zip › Source Data_EV figuresAndAppendix/EV2/Parasite18_231204_244_30MIN_1min_2002.nd2 - 231204_244_30MIN_1min_2002.nd2 (series 4)-1.tif]

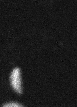

Supplement: Supplementary file 7 — EV Figures source data [file 44319_2024_280_MOESM7_ESM.zip › Source Data_EV figuresAndAppendix/EV2/Parasite23_231212_244_19MIN_1min_.nd2 - 231212_244_19MIN_1min_.nd2 (series 2)-1.tif]

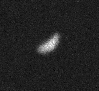

Supplement: Supplementary file 7 — EV Figures source data [file 44319_2024_280_MOESM7_ESM.zip › Source Data_EV figuresAndAppendix/EV2/Parasite24_231212_244_19MIN_1min_.nd2 - 231212_244_19MIN_1min_.nd2 (series 3)-1.tif]

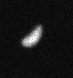

Supplement: Supplementary file 7 — EV Figures source data [file 44319_2024_280_MOESM7_ESM.zip › Source Data_EV figuresAndAppendix/EV2/Parasite26_231212_244_19MIN_1min_.nd2 - 231212_244_19MIN_1min_.nd2 (series 4)-1.tif]

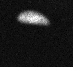

Supplement: Supplementary file 7 — EV Figures source data [file 44319_2024_280_MOESM7_ESM.zip › Source Data_EV figuresAndAppendix/EV2/Parasite27_231212_244_19MIN_1min_.nd2 - 231212_244_19MIN_1min_.nd2 (series 5)-1.tif]

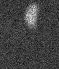

Supplement: Supplementary file 7 — EV Figures source data [file 44319_2024_280_MOESM7_ESM.zip › Source Data_EV figuresAndAppendix/EV2/Parasite13_231204_244_30MIN_1min_2002.nd2 - 231204_244_30MIN_1min_2002.nd2 (series 1)-1.tif]

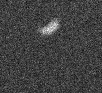

Supplement: Supplementary file 7 — EV Figures source data [file 44319_2024_280_MOESM7_ESM.zip › Source Data_EV figuresAndAppendix/EV2/Parasite16_231204_244_30MIN_1min_2002.nd2 - 231204_244_30MIN_1min_2002.nd2 (series 2)-1.tif]

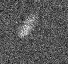

Supplement: Supplementary file 7 — EV Figures source data [file 44319_2024_280_MOESM7_ESM.zip › Source Data_EV figuresAndAppendix/EV2/Parasite12_231204_244_30MIN_1min_2002.nd2 - 231204_244_30MIN_1min_2002.nd2 (series 1)-1.tif]

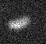

Supplement: Supplementary file 7 — EV Figures source data [file 44319_2024_280_MOESM7_ESM.zip › Source Data_EV figuresAndAppendix/EV2/Parasite9_231129_23MIN_1min.nd2 - 231129_23MIN_1min.nd2 (series 3)-1.tif]

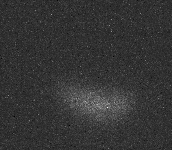

Supplement: Supplementary file 7 — EV Figures source data [file 44319_2024_280_MOESM7_ESM.zip › Source Data_EV figuresAndAppendix/EV2/Parasite2_from video on manuscript_211013_GFPRNF_RHtomato_1hmin_30s008-1.tif]

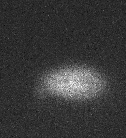

Supplement: Supplementary file 7 — EV Figures source data [file 44319_2024_280_MOESM7_ESM.zip › Source Data_EV figuresAndAppendix/EV2/Parasite4_211013_GFPRNF_RHtomato_30min_2min001.nd2 - 211013_GFPRNF_RHtomato_30min_2min001.nd2 (series 04)-1.tif]

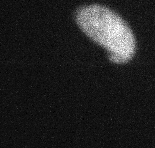

Supplement: Supplementary file 7 — EV Figures source data [file 44319_2024_280_MOESM7_ESM.zip › Source Data_EV figuresAndAppendix/EV2/Parasite3_211013_GFPRNF_RHtomato_30min_2min001.nd2 - 211013_GFPRNF_RHtomato_30min_2min001.nd2 (series 04)-1.tif]

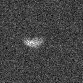

Supplement: Supplementary file 7 — EV Figures source data [file 44319_2024_280_MOESM7_ESM.zip › Source Data_EV figuresAndAppendix/EV2/Parasite21_231204_244_30MIN_1min_2002.nd2 - 231204_244_30MIN_1min_2002.nd2 (series 5)-1.tif]

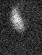

Supplement: Supplementary file 7 — EV Figures source data [file 44319_2024_280_MOESM7_ESM.zip › Source Data_EV figuresAndAppendix/EV2/Parasite5_231129_23MIN_1min.nd2 - 231129_23MIN_1min.nd2 (series 1)-1.tif]

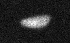

Supplement: Supplementary file 7 — EV Figures source data [file 44319_2024_280_MOESM7_ESM.zip › Source Data_EV figuresAndAppendix/EV2/Parasite14_231204_244_30MIN_1min_2002.nd2 - 231204_244_30MIN_1min_2002.nd2 (series 1)-1.tif]

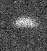

Supplement: Supplementary file 7 — EV Figures source data [file 44319_2024_280_MOESM7_ESM.zip › Source Data_EV figuresAndAppendix/EV2/Parasite8_231129_23MIN_1min.nd2 - 231129_23MIN_1min.nd2 (series 2)-1.tif]

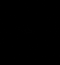

Supplement: Supplementary file 7 — EV Figures source data [file 44319_2024_280_MOESM7_ESM.zip › Source Data_EV figuresAndAppendix/EV2/Parasite20_231204_244_30MIN_1min_2002.nd2 - 231204_244_30MIN_1min_2002.nd2 (series 5)-1.tif]

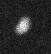

Supplement: Supplementary file 7 — EV Figures source data [file 44319_2024_280_MOESM7_ESM.zip › Source Data_EV figuresAndAppendix/EV2/Parasite10_231129_23MIN_1min.nd2 - 231129_23MIN_1min.nd2 (series 4)-1.tif]
