# Supplementary material for: Recognition of phylogenetically diverse pathogens through enzymatically amplified recruitment of RNF213
Source: EMBO Rep. 2024 Oct 7;25(11):4979–5005. doi: 10.1038/s44319-024-00280-w (PMC11549300; doi:10.1038/s44319-024-00280-w)
Supplement: Supplementary file 12 — Expanded View Figures [file 44319_2024_280_MOESM12_ESM.pdf]

Expanded View Figures

Figure EV1. RNF213 accumulates on phylogenetically distant pathogens.

(**A**) Confocal micrographs of HeLa cells infected with *S.* Typhimurium for 4 h and *L. monocytogenes* or *T. gondii* infected for 6 h. Cells were stained with anti-RNF213 antibody. Scale bar 20 µm (magnification box scale bar; 5 µm). (**B**) Confocal micrographs representative of quantifications shown in Fig. 1E. MEFs stimulated with IFNγ as indicated, infected with Tomato-expressing *T. gondii* Type I RH or Type II Pru for 1 h and stained with anti-ubiquitin (FK2) antibody and DAPI. Regions marked with white borders in the main images are shown magnified on the right. Scale bar 80 µm (magnification box, scale bar; 20 µm). Source data are available online for this figure.

 © The Author(s)

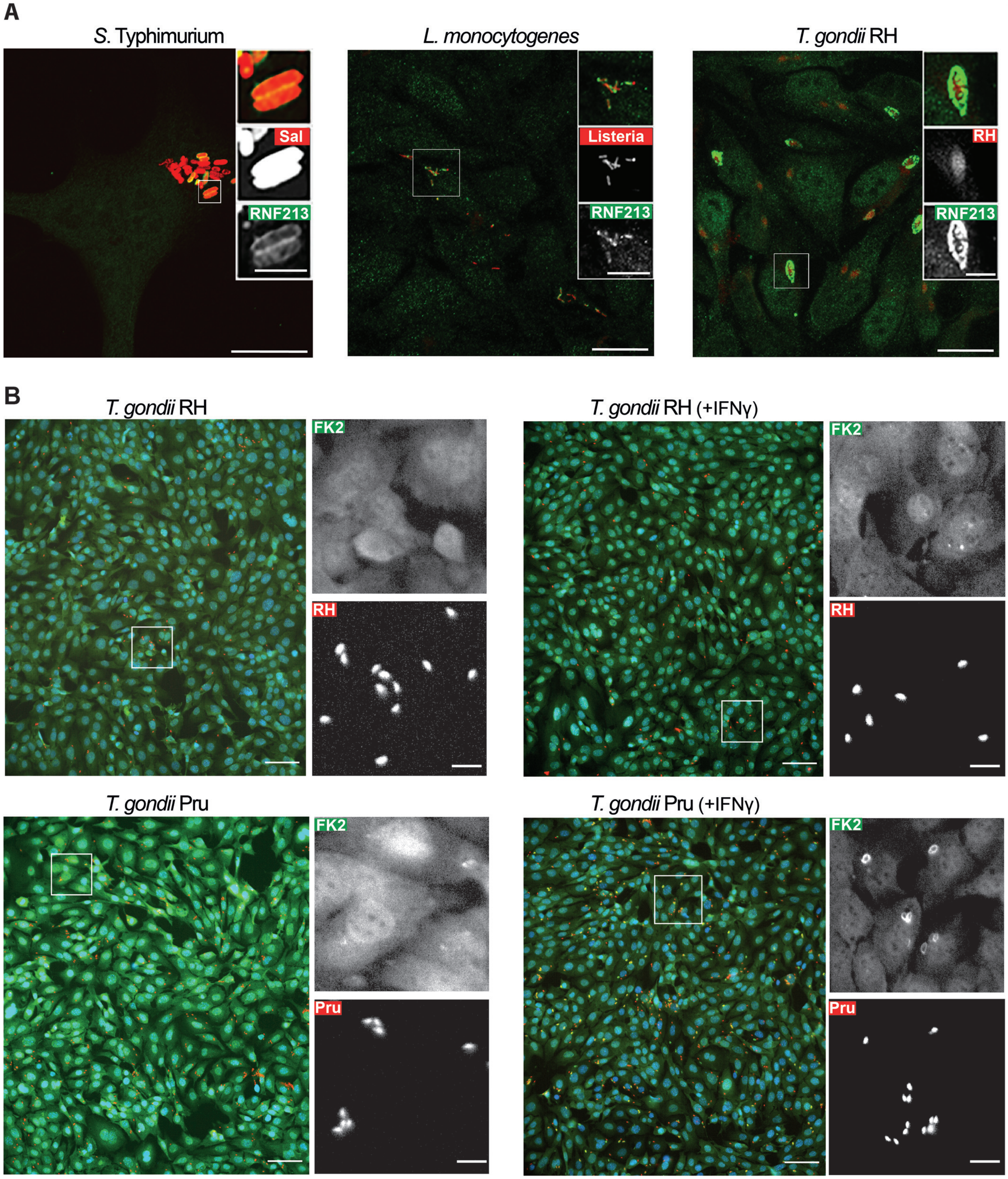
A
S. Typhimurium
Sal
RNF213
L. monocytogenes
Listeria
RNF213
T. gondii RH
RH
RNF213
B
T. gondii RH
FK2
RH
T. gondii RH (+IFNγ)
FK2
RH
T. gondii Pru
FK2
Pru
T. gondii Pru (+IFNγ)
FK2
Pru

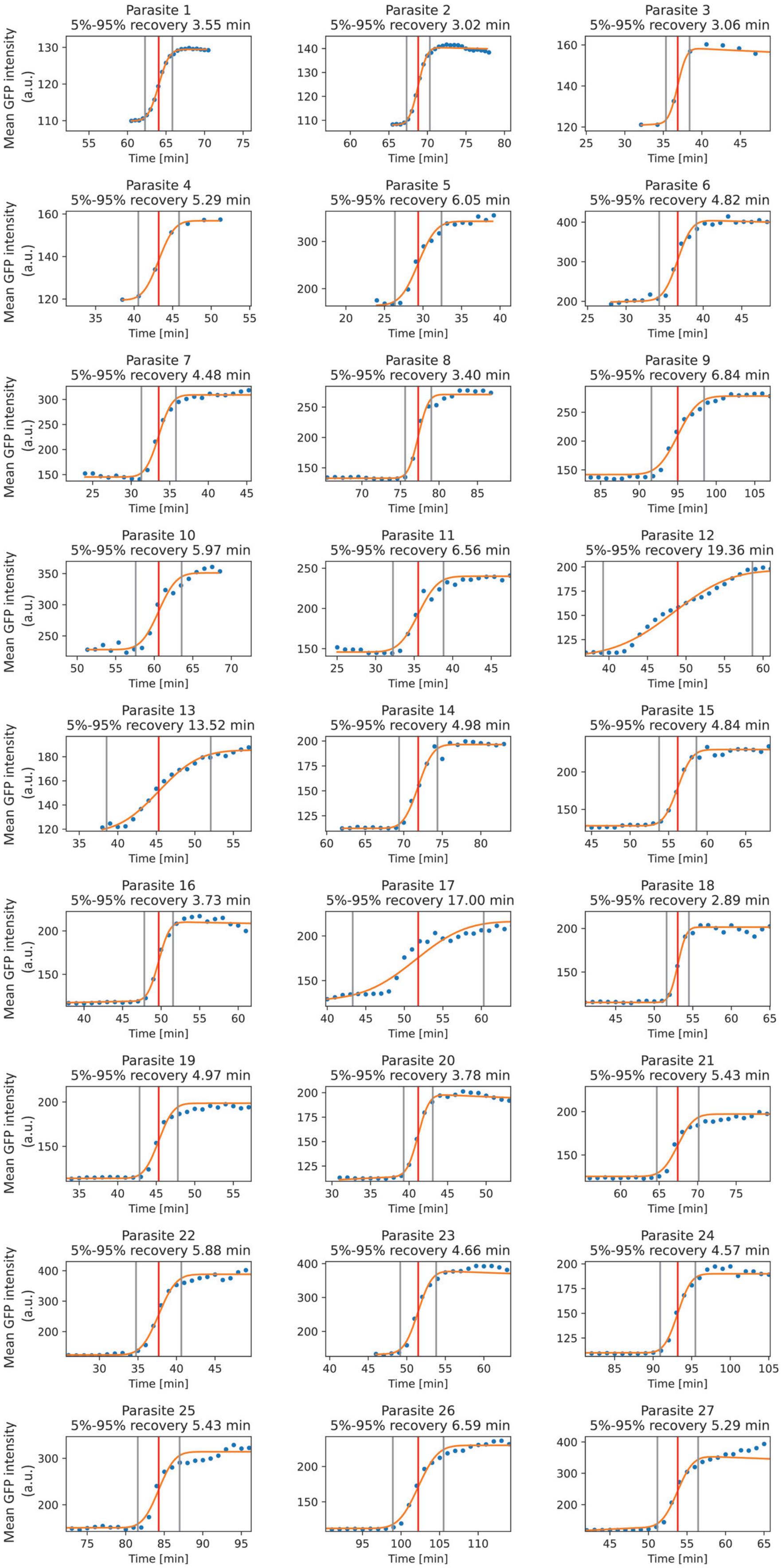
Parasite 1
5%-95% recovery 3.55 min
Parasite 2
5%-95% recovery 3.02 min
Parasite 3
5%-95% recovery 3.06 min
Parasite 4
5%-95% recovery 5.29 min
Parasite 5
5%-95% recovery 6.05 min
Parasite 6
5%-95% recovery 4.82 min
Parasite 7
5%-95% recovery 4.48 min
Parasite 8
5%-95% recovery 3.40 min
Parasite 9
5%-95% recovery 6.84 min
Parasite 10
5%-95% recovery 5.97 min
Parasite 11
5%-95% recovery 6.56 min
Parasite 12
5%-95% recovery 19.36 min
Parasite 13
5%-95% recovery 13.52 min
Parasite 14
5%-95% recovery 4.98 min
Parasite 15
5%-95% recovery 4.84 min
Parasite 16
5%-95% recovery 3.73 min
Parasite 17
5%-95% recovery 17.00 min
Parasite 18
5%-95% recovery 2.89 min
Parasite 19
5%-95% recovery 4.97 min
Parasite 20
5%-95% recovery 3.78 min
Parasite 21
5%-95% recovery 5.43 min
Parasite 22
5%-95% recovery 5.88 min
Parasite 23
5%-95% recovery 4.66 min
Parasite 24
5%-95% recovery 4.57 min
Parasite 25
5%-95% recovery 5.43 min
Parasite 26
5%-95% recovery 6.59 min
Parasite 27
5%-95% recovery 5.29 min
Mean GFP intensity (a.u.)
Time [min]

◀ **Figure EV2. Kinetics of GFP-RNF213 accumulation on individual *T. gondii* vacuoles.**

Kinetics of GFP-RNF213 accumulation on *T. gondii* vacuoles. Dots represent the measured GFP intensity. An error function was fitted (orange line) to calculate the 5% and 95% of maximum coat formation (vertical grey lines) as well as the midpoint (vertical red line). Time required for coat completion (5% to 95% of maximum fluorescence) is annotated above the graphs. n = 6 biological experiments; parasites 1 and 2 from experiment 1; parasites 3 and 4 from experiment 2; parasites 5 to 10 from experiment 3; parasite 11 from experiment 4; parasites 12 to 21 from experiment 5; parasites 22 to 27 from experiment 6. Acquisition started at 60 min, 30 min, 23 min, 24 min, 30 min and 19 min p.i, respectively. Experiments 1 and 2 were acquired on a Nikon iSIM swept field high speed inverted microscope with a 100X super resolution Apo TIRF oil objective, experiments 3 to 6 on a Nikon X1 Spinning Disk inverted microscope with a 40x/1.3NA Oil lens. Source data are available online for this figure.

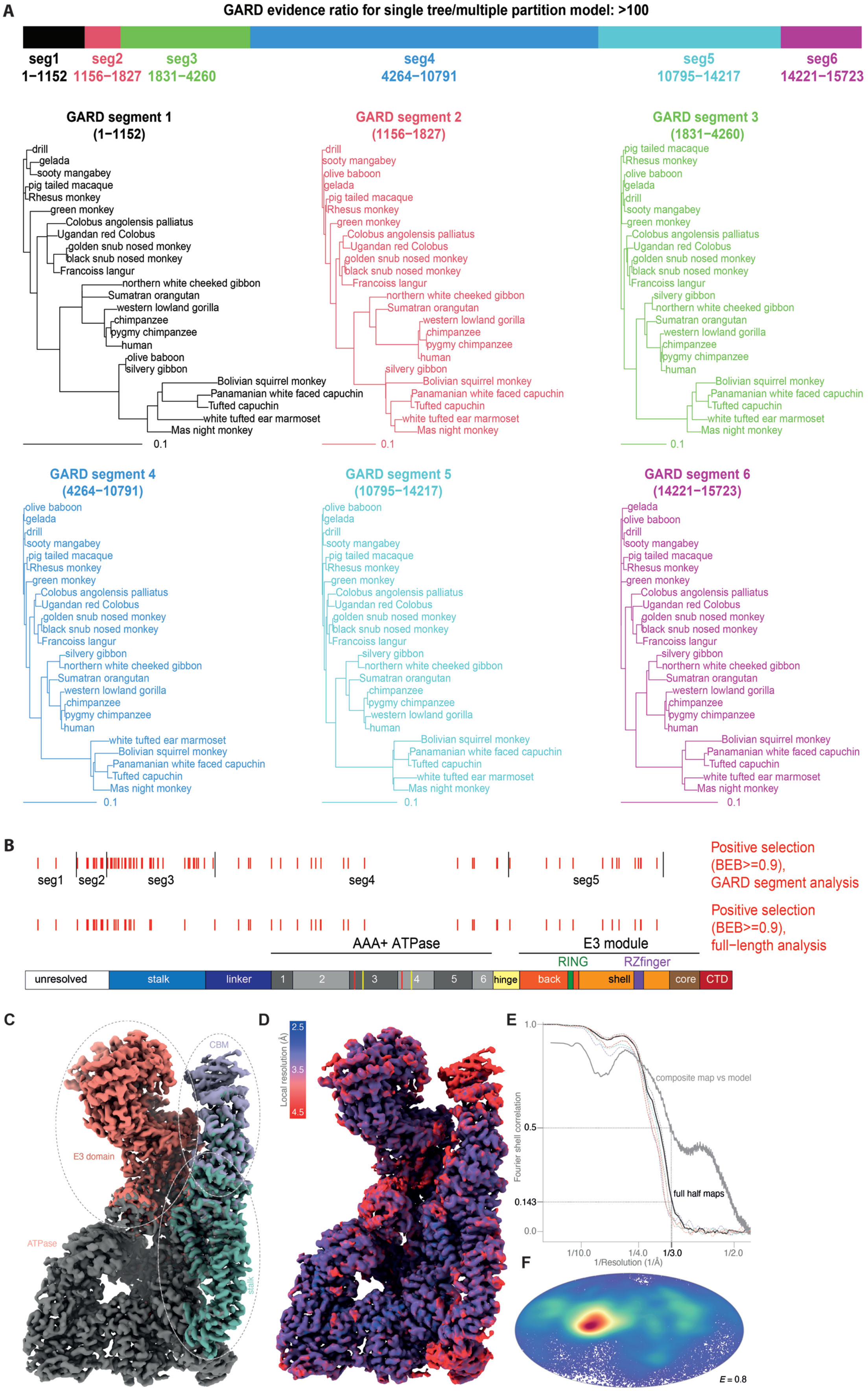
A
GARD evidence ratio for single tree/multiple partition model: >100
seg1 1–1152
seg2 1156–1827
seg3 1831–4260
seg4 4264–10791
seg5 10795–14217
seg6 14221–15723
GARD segment 1 (1–1152)
GARD segment 2 (1156–1827)
GARD segment 3 (1831–4260)
GARD segment 4 (4264–10791)
GARD segment 5 (10795–14217)
GARD segment 6 (14221–15723)
0.1
B
seg1 seg2 seg3 seg4 seg5
Positive selection (BEB>=0.9), GARD segment analysis
Positive selection (BEB>=0.9), full–length analysis
AAA+ ATPase
E3 module
RING
RZfinger
unresolved
stalk
linker
1 2 3 4 5 6
hinge
back
shell
core
CTD
C
CBM
E3 domain
ATPase
stalk
D
Local resolution (Å)
2.5
3.5
4.5
E
composite map vs model
full half maps
Fourier shell correlation
1.0
0.5
0.143
0.0
1/10.0 1/4.0 1/3.0 1/2.0
1/Resolution (1/Å)
F
E = 0.8

◀ **Figure EV3. Positive selection in GARD-identified RNF213 segments and cryo-EM analysis of RNF213.**

(**A**) The GARD algorithm detects evidence of possible recombination among RNF213 simian primate orthologs, with an evidence ratio of >100. Schematic of the 6 segments identified by GARD, and the phylogenies for each segment according to GARD. Differences between segment topologies are mostly subtle, in basal branches of the tree. (**B**) Repeat of codeml analysis, performed individually on each of the 6 GARD segment alignments. Segments 1–5 all showed evidence of positive selection (Appendix Table S1). Indicated with red tick marks are residues with high probability (Bayes Empirical Bayes posterior probability ≥0.9) of evolving under positive selection identified by GARD segment and full-length analysis, as indicated. Analysing the 6 GARD segments separately appears to increase the statistical power of codeml to identify rapidly evolving sites: across the 5 segments, a total of 86 sites have ≥90% posterior probability (Bayes Empirical Bayes) of evolving under positive selection, in contrast to 59 sites when analyzing the full-length alignment. (**C**) Composite map of RNF213 created by local refinement of individual color-coded domains. (**D**) Composite map of RNF213 coloured by local resolution, calculated using ResMap (Kucukelbir et al, 2014). (**E**) Fourier shell correlation between the two consensus half-maps (solid black line), and between the composite map and the refined atomic model (solid grey line), is plotted as a function of resolution. The Fourier shell correlations for each pair of locally refined half maps are shown with the dashed lines, coloured according to the domains in (**A**). (**F**) Orientation distribution of the RNF213 particles used in the consensus refinement shown on a Mollweide projection plot, coloured in blue to red from low to high density, and has an efficiency E = 0.8, calculated using cryoEF (Naydenova and Russo, 2017).

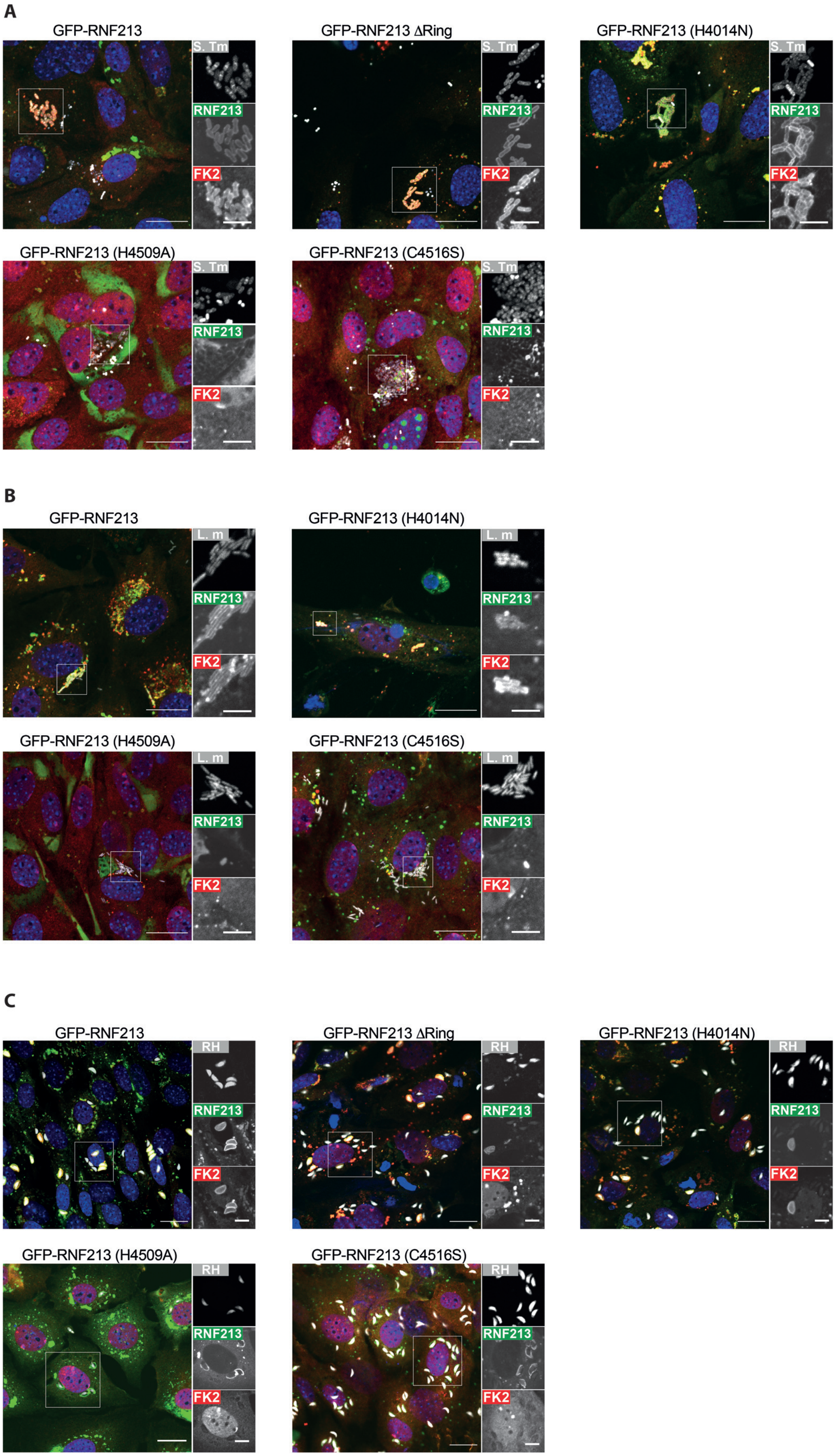
A
GFP-RNF213
GFP-RNF213 ΔRing
GFP-RNF213 (H4014N)
GFP-RNF213 (H4509A)
GFP-RNF213 (C4516S)
S. Tm
RNF213
FK2
B
GFP-RNF213
GFP-RNF213 (H4014N)
GFP-RNF213 (H4509A)
GFP-RNF213 (C4516S)
L. m
RNF213
FK2
C
GFP-RNF213
GFP-RNF213 ΔRing
GFP-RNF213 (H4014N)
GFP-RNF213 (H4509A)
GFP-RNF213 (C4516S)
RH
RNF213
FK2

© The Author(s)

Figure EV4. Enzymatic activity in RNF213 is required for coat formation on pathogens.

Confocal micrographs representative of quantifications shown in Fig. 4D–F. RNF213^{KO} MEFs complemented with the indicated GFP-RNF213 alleles and stained with anti-ubiquitin antibody (FK2) and DAPI at 4 h post-infection with mCherry-expressing *S. Typhimurium* (**A**), 6 h post-infection with mCherry-expressing *L. monocytogenes ΔActA* (**B**) and 1 h post-infection with Tomato-expressing *T. gondii* RH Type I strain (**C**). Regions marked with white borders in the main images are shown magnified on the right. Scale bar, 20 μm (magnification box scale bar, 10 μm). Source data are available online for this figure.

© The Author(s)
